# Supplementary material for: Operating at the extreme: estimating the upper yield boundary of winter wheat production in commercial practice
Source: R Soc Open Sci. 2020 Apr 22;7(4):191919. doi: 10.1098/rsos.191919 (PMC7211843; doi:10.1098/rsos.191919)
Supplement: Supplementary Material [file rsos191919supp2.pdf]

Supplementary Material document to: Operating at the extreme: estimating the upper yield boundary of winter wheat production in commercial practice

Emily G. Mitchell[(1)], Neil M.J. Crout[(2)], Paul Wilson[(2)],
Andrew T.A. Wood[(4)] & Gilles Stupfler[(1,3)]

[(1)] School of Mathematical Sciences, University of Nottingham, Nottingham NG7 2RD, UK
[(2)] School of Biosciences, University of Nottingham, Sutton Bonington LE12 5RD, UK
[(3)] Univ Rennes, Ensai, CNRS, CREST - UMR 9194, F-35000 Rennes, France
[(4)] RSFAS, Australian National University, ACT 2601 Australia

Sections A and B respectively give theoretical and practical backing for our way of selecting the data. Section C presents an alternative way to construct confidence intervals for model parameters and high yields, using the profile likelihood method.

A A theoretical justification for our selection of data

Our construction of the data relies on taking the maximum yield for each farm, when this farm has returned data over multiple years. We give here justification that this way of selecting the data allows to make inference that is consistent with our initial aim, which was to estimate the maximum value of a one-year yield. Let Y be a random variable having the distribution of the one-year yield for a farm (over the whole of England and Wales). For a given farm in our sample, yields are recorded over N years, where $N \in \{1, \ldots, 10\}$ is a discrete random variable, and we take the maximum such yield as an individual data point. In other words, our data is made of $n = 1536$ data points whose distribution is that of $X = \max_{1 \le i \le N} Y_i$, where the Y_i are independent copies of Y. We suppose in what follows that N is independent of Y and thus of X.

It should, first, be clear that X and Y have the same support and in particular the same right endpoint x^*, so that making inference about the right endpoint using a sample from X rather than Y is indeed possible. We then have, for any $t < x^*$,

$$\forall y > 0, \ \mathbb{P}\left(X - t \le y \,|\, X > t\right) = \frac{\mathbb{P}(\max_{1 \le i \le N} Y_i \le y + t) - \mathbb{P}(\max_{1 \le i \le N} Y_i \le t)}{1 - \mathbb{P}(\max_{1 \le i \le N} Y_i \le t)}.$$

By independence of N and the Y_i, the numerator above can be rewritten

$$\begin{aligned} & \mathbb{P}\left(\max_{1\leq i\leq N} Y_i \leq y+t\right) - \mathbb{P}\left(\max_{1\leq i\leq N} Y_i \leq t\right) \\ = & \sum_{m=1}^{10}\left[\mathbb{P}\left(\max_{1\leq i\leq m} Y_i \leq y+t\right) - \mathbb{P}\left(\max_{1\leq i\leq m} Y_i \leq t\right)\right]\mathbb{P}(N=m) \\ = & \sum_{m=1}^{10}\left[\{\mathbb{P}\left(Y\leq y+t\right)\}^m - \{\mathbb{P}\left(Y\leq t\right)\}^m\right]\mathbb{P}(N=m) \\ = & \left[\mathbb{P}\left(Y\leq y+t\right) - \mathbb{P}\left(Y\leq t\right)\right]\sum_{m=1}^{10}\mathbb{P}(N=m)\sum_{j=0}^{m-1}\{\mathbb{P}\left(Y\leq y+t\right)\}^j\{\mathbb{P}\left(Y\leq t\right)\}^{m-1-j}. \end{aligned}$$

When t is high, and regardless of the value of $y>0$, $\mathbb{P}\left(Y\leq y+t\right)$ and $\mathbb{P}\left(Y\leq t\right)$ are close to 1; in other words, we have

$$\begin{aligned} \mathbb{P}\left(\max_{1\leq i\leq N} Y_i \leq y+t\right) - \mathbb{P}\left(\max_{1\leq i\leq N} Y_i \leq t\right) & \approx \left[\mathbb{P}\left(Y\leq y+t\right) - \mathbb{P}\left(Y\leq t\right)\right]\sum_{m=1}^{10} m\,\mathbb{P}(N=m) \\ & = \left[\mathbb{P}\left(Y\leq y+t\right) - \mathbb{P}\left(Y\leq t\right)\right]\mathbb{E}(N) \end{aligned}$$

when t is high, for any $y>0$. Similarly

$$1-\mathbb{P}\left(\max_{1\leq i\leq N} Y_i \leq t\right) \approx \left[1-\mathbb{P}\left(Y\leq t\right)\right]\mathbb{E}(N)$$

when t is high, and therefore

$$\begin{aligned} \forall y>0,\ \mathbb{P}\left(X-t\leq y\,|\,X>t\right) & \approx \frac{\mathbb{P}\left(Y\leq y+t\right)-\mathbb{P}\left(Y\leq t\right)}{1-\mathbb{P}\left(Y\leq t\right)} \\ & = \mathbb{P}\left(Y-t\leq y\,|\,Y>t\right). \end{aligned}$$

This implies that the generalised Pareto model appropriate to model the right tail of the one-year yield Y is also a sensible model for the right tail of our multiple-year maximum X. In terms of bias, using multiple-year data is actually intuitively better than single-year data, since their values will tend to be larger and therefore closer to the true value of the right endpoint.

B Sensitivity of our results to data selection

The validity of the technical argument in Section A rests on the assumption of independent and identically distributed yields across time and space. In our context where only ten years of data are available, evaluating precisely the amount of spatial and/or temporal dependence in the data is a very difficult task. Modelling spatial dependence would require a careful model of the physical processes underpinning wheat growth; in addition to the level of agricultural input, a sensible comprehensive model would need to consider the local chemical composition of soil, which is very difficult to gather and clearly not a simple function of location, as well as (at least!) the behaviour of rainfall, temperature and solar radiation across the regions of interest. State-of-the-art models, such as the model of Kern *et al.* (2018), are typically regression models with a view on modelling

average yield levels as a function of physical covariates. As a first step in the analysis of high yields, it is, we believe, advisable to start by a simpler model, which is why we adopted a model where yields are considered spatially independent.

We can, however, get an idea of the influence of temporal dependence through the use of alternative approaches to data selection, for example by working solely with one year of data, or by randomly choosing a year of data for each farm. To be more specific, we considered the following samples of yield:

- Yields for the year 2009/2012/2015 only,
- Yields for a single randomly selected year of data for each farm,
- Maximum yields over a randomly selected block of 5 years of data.

The rationale for these choices was that we wanted to test whether selecting an average year for yield (2009), a poor year for yield (2012), a good year for yield (2015), or yields over a randomly selected smaller number of years had significant consequences on our estimates. [That 2009, 2012 and 2015 can be considered "average", "poor" and "good" years respectively for yield can be inferred from the boxplots given in Figure 1 of the main paper.] Let us repeat here that our final objective is **to estimate the best possible yield under current growing conditions**. As a consequence, **we are not interested in the evolution of maximum yield through time** and it is thus meaningful to compare these right endpoint estimates: even though the underlying parameters of the generalised Pareto model may well change when data is constructed by taking maxima over several years (if there is temporal dependence), the maximum possible value of yield, which is our target here, stays unaffected.

Numerical results are reported in Table 1, and illustrations of the finite-sample behaviour of the associated extreme value index estimator are given in Figures 1 and 2 along with the selected sample fraction in each case. It can be seen in this table that the obtained endpoint estimates for years 2009 and 2012 lie outside the confidence interval for the endpoint calculated using our original construction of the data. This, in our view, should be expected because 2009 and 2012 were respectively average and poor years for yield, and a selection of yield data that is not consistent with our objective of inferring the best possible yield cannot be expected to give sensible results. This is just as in the problem of estimating records in athletics: to do so, it makes sense to consider data made of the best performances of the best athletes (see Einmahl and Magnus, 2008), rather than work on the best performances of average or poor athletes. The estimate based on a single, randomly chosen year of data is also markedly lower than our initial estimate (although perhaps not significantly so at the 95% level). By contrast, the obtained endpoint estimates for year 2015 or for the data made of the best yields across 5 randomly selected years are in line with our initial estimate (if slightly higher but not significantly so), thus illustrating that if an effort is made to select data relevant to our purposes, the actual selection method does not have a strong influence on maximum yield estimates.

**Year**	n	k	t	Shape estimate $\hat{\gamma}$	Scale estimate $\hat{\sigma}$	$\hat{x}^* = t - \hat{\sigma}/\hat{\gamma}$
All years combined	1536	250	10.69	-0.11 $(-0.22, 0.00)$	0.76 $(0.65, 0.91)$	17.60 $(14.02, 23.75)$
2009 only	676	140	9.44	-0.22 $(-0.35, -0.096)$	0.85 $(0.70, 1.08)$	13.24 $(12.31, 14.86)$
2012 only	721	250	7.16	-0.17 $(-0.28, -0.07)$	1.09 $(0.93, 1.29)$	13.41 $(11.88, 16.40)$
2015 only	674	100	10.99	-0.095 $(-0.27, 0.08)$	0.73 $(0.57, 0.99)$	18.62 $(14.02, 31.40)$
One randomly chosen year per farm	1536	200	9.96	-0.22 $(-0.33, -0.12)$	0.98 $(0.83, 1.19)$	14.32 $(13.28, 15.88)$
Five randomly chosen years combined	1314	230	10.69	-0.093 $(-0.21, 0.02)$	0.74 $(0.63, 0.89)$	18.59 $(14.02, 27.56)$

Table 1: Maximum yield level estimates $\hat{x}^*$ for our original data set and the data selected in alternative ways, along with a summary of sample sizes, threshold choices, shape estimates $\hat{\gamma}$ and scale estimates $\hat{\sigma}$. Numbers in brackets next to shape, scale and maximum yield estimates represent approximate 95% confidence intervals.

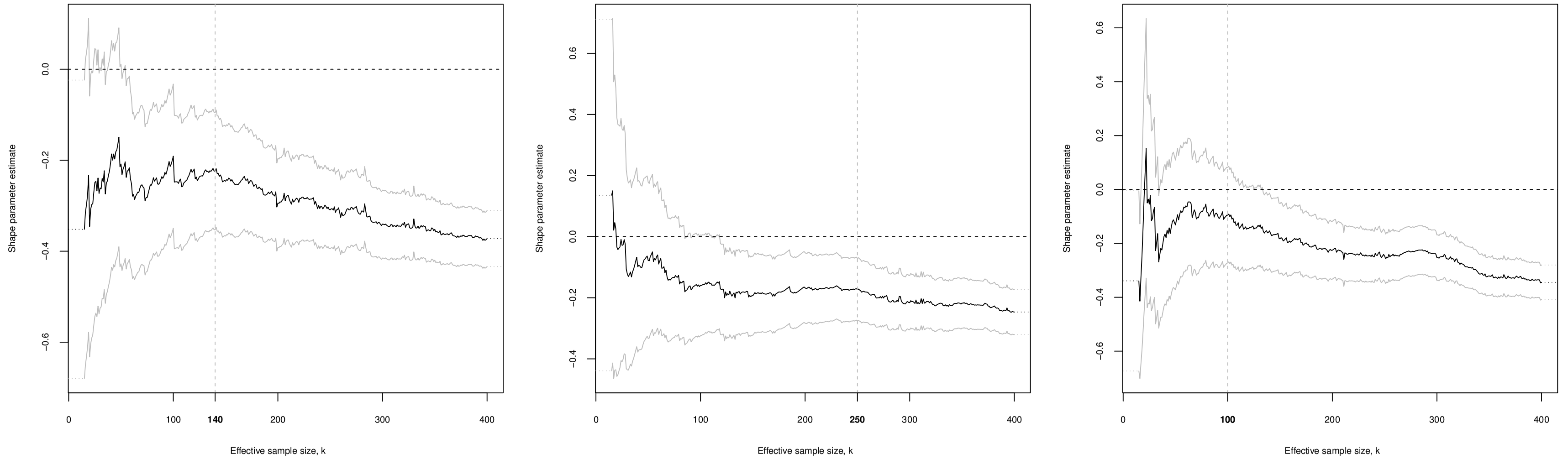

Figure 1: ML estimates of γ, for years 2009 (left), 2012 (middle), and 2015 (right) only.

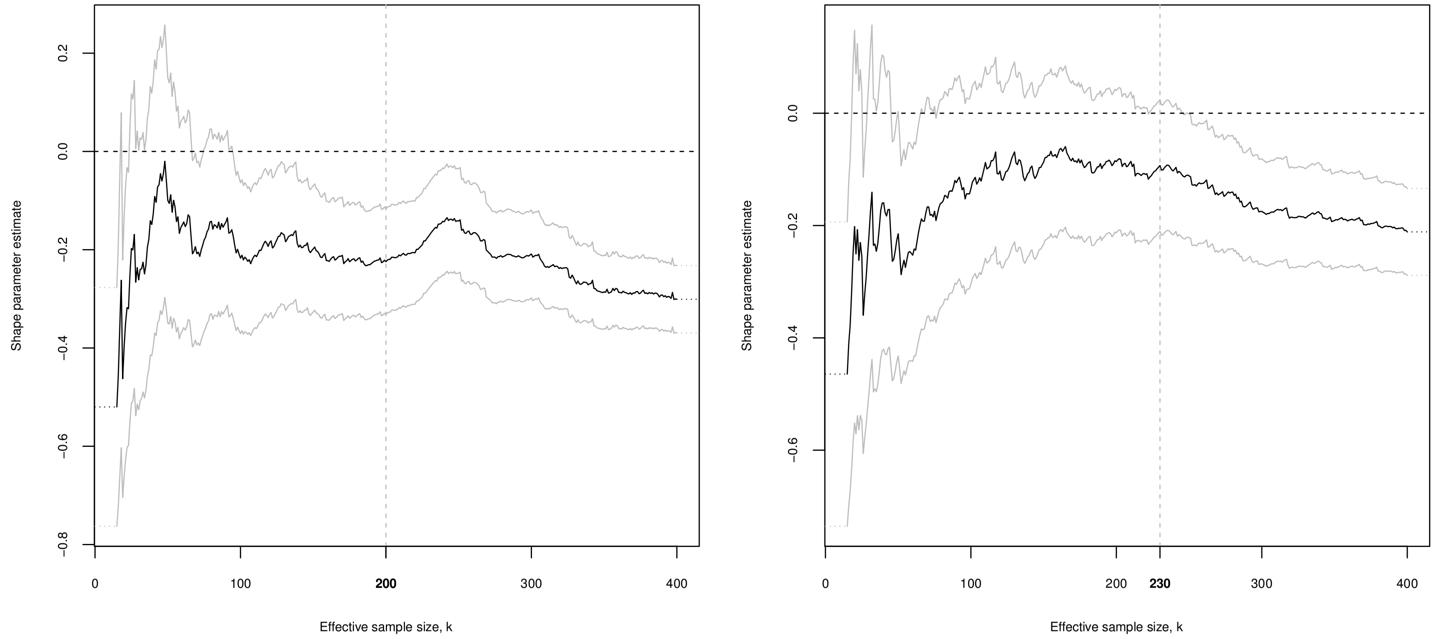

Figure 2: ML estimates of γ, for data constructed by choosing a single randomly selected year for each farm (left) and the maximum yield over a randomly selected block of 5 years of data (right).

C An alternative view on confidence intervals using profile likelihood

Figures 3 and 4 on the next page provide 95% profile likelihood based confidence intervals for the shape parameter and for extreme return levels of wheat yield in a generalised Pareto model, using the full data set. The method for calculating such confidence intervals is explained in Chapters 3 and 4 of Coles (2001). The actual calculations are carried out numerically using the `gpd.fit` and `gpd.prof` routines part of the R package `ismev`, put together by Heffernan and Stephenson and maintained by Gilleland (2018).

It can be seen in Figure 3 that the 95% profile likelihood confidence interval for the shape parameter contains 0. In such a situation, the profile likelihood function becomes flatter as the return level m increases, and the upper bound of the confidence interval tends to infinity as $m \to \infty$. This is illustrated in Figure 4, where one can see that the upper bound of the confidence interval for the return level escapes to the right of the plot as m increases.

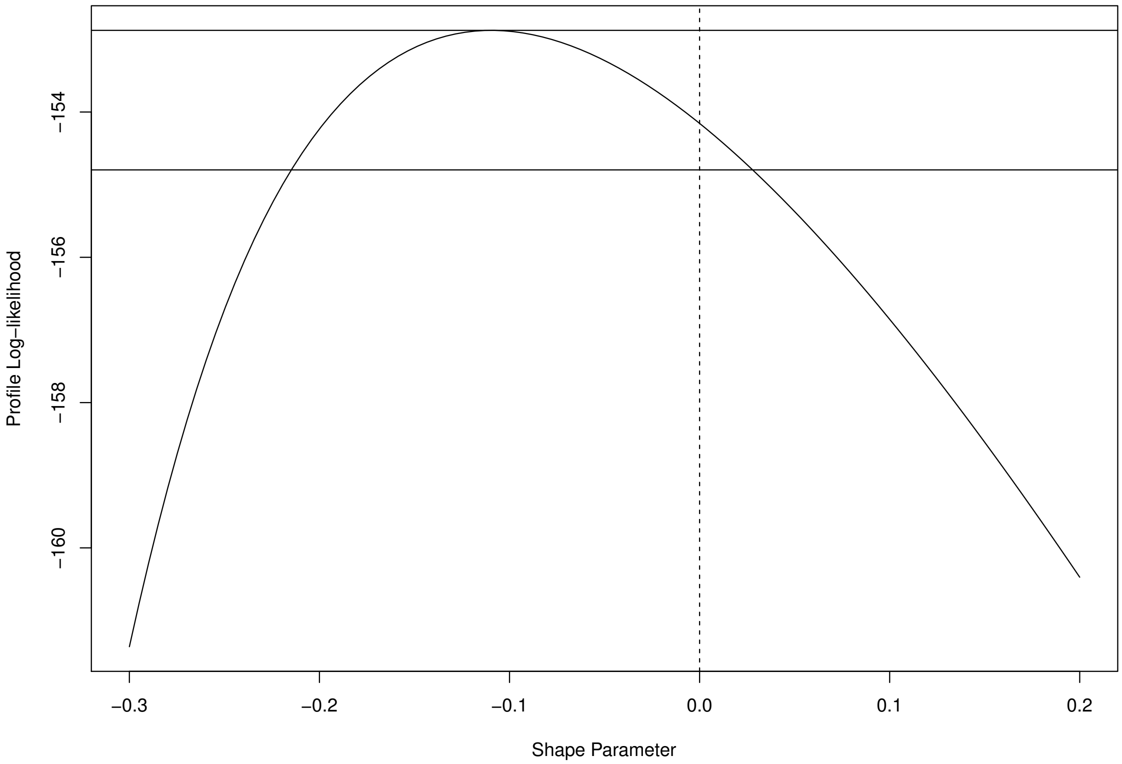

Figure 3: Full yield data set of size $n = 1536$, graph of the profile likelihood function for the shape parameter γ. The two intersections of the concave full line with the second-from-top horizontal full line define the 95% profile likelihood confidence interval for γ. The vertical dashed line is the line $\gamma = 0$.

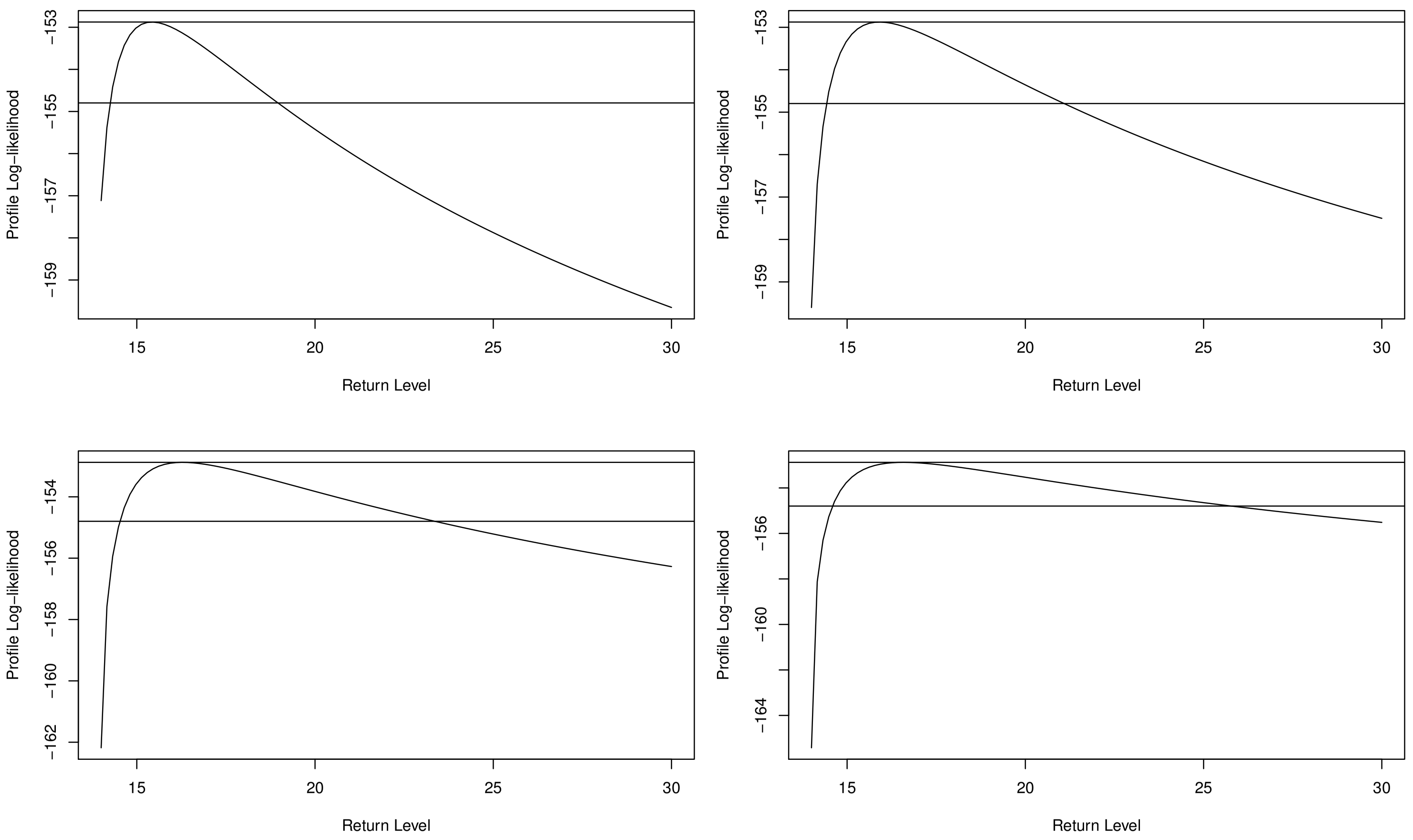

Figure 4: Full yield data set of size $n = 1536$, graphs of the profile likelihood functions for return levels $m \in \{n/10, n, 10\,n, 100\,n\}$. Top left: return level with exceedance probability $10/n$, top right: return level with exceedance probability $1/n$, bottom left: return level with exceedance probability $1/(10n)$, bottom right: return level with exceedance probability $1/(100n)$. In each graph, the two intersections of the concave full line with the second-from-top horizontal full line define the 95% profile likelihood confidence interval.

References

Coles, S.G. (2001). *An Introduction to Statistical Modeling of Extreme Values.* Springer-Verlag, London.

Einmahl, J.H.J. and Magnus, J.R. (2008). Records in athletics through extreme-value theory, *Journal of the American Statistical Association* **103**(484): 1382–1391.

Heffernan, J.E., Stephenson, A.G. and Gilleland, E. (2018). *ismev: An Introduction to Statistical Modeling of Extreme Values*, R package version 1.42.

Kern, A., Barcza, Z., Marjanović, H., Árendás, T., Fodor, N., Bónis, P., Bognár, P. and Lichtenberger, J. (2018). Statistical modelling of crop yield in Central Europe using climate data and remote sensing vegetation indices, *Agricultural and Forest Meteorology* **260–261**: 300–320.
